# Beyond rhythm – a framework for understanding the frequency spectrum of neural activity

**DOI:** 10.3389/fnsys.2023.1217170

**Published:** 2023-08-31

**Authors:** Quentin Perrenoud, Jessica A. Cardin

**Affiliations:** Department of Neuroscience, Yale School of Medicine, Kavli Institute for Neuroscience, Wu Tsai Institute, New Haven, CT, United States

**Keywords:** Fourier - spectrometry, local field potential (LFP), oscillation, neural circuits, rhythm, shot noise analysis

## Abstract

Cognitive and behavioral processes are often accompanied by changes within well-defined frequency bands of the local field potential (LFP i.e., the voltage induced by neuronal activity). These changes are detectable in the frequency domain using the Fourier transform and are often interpreted as neuronal oscillations. However, aside some well-known exceptions, the processes underlying such changes are difficult to track in time, making their oscillatory nature hard to verify. In addition, many non-periodic neural processes can also have spectra that emphasize specific frequencies. Thus, the notion that spectral changes reflect oscillations is likely too restrictive. In this study, we use a simple yet versatile framework to understand the frequency spectra of neural recordings. Using simulations, we derive the Fourier spectra of periodic, quasi-periodic and non-periodic neural processes having diverse waveforms, illustrating how these attributes shape their spectral signatures. We then show how neural processes sum their energy in the local field potential in simulated and real-world recording scenarios. We find that the spectral power of neural processes is essentially determined by two aspects: (1) the distribution of neural events in time and (2) the waveform of the voltage induced by single neural events. Taken together, this work guides the interpretation of the Fourier spectrum of neural recordings and indicates that power increases in specific frequency bands do not necessarily reflect periodic neural activity.

## Introduction

The Fourier transform, or analogous methods, is routinely used to analyze the frequency content (i.e., the spectrum) of neural activity (Penttonen and Buzsáki, 2003; Bruns, 2004). Frequency spectra are highly sensitive to changes in the processes driving a signal (Percival and Walden, 1993). They can thus be used to detect subtle variations in the dynamics of the brain’s electric fields during attention, memory formation or retrieval, locomotion, and motor responses (Gray et al., 1989; O’Keefe and Recce, 1993; Skaggs et al., 1996; Fries et al., 2001; Girardeau et al., 2009; Niell and Stryker, 2010; Bosman et al., 2012; Vinck et al., 2015; Chen et al., 2017; Veit et al., 2017; Uran et al., 2022). However, the behavior of frequency spectra is often counterintuitive. For instance, the finite nature of recordings induces distortion of their spectral content (Percival and Walden, 1993). Furthermore, relating frequency spectra to continuously evolving neural processes is a complex problem. As a result, the interpretation of the frequency spectrum of neural activity is not straightforward.

Increased spectral power within some frequency bands is often believed to reflect oscillations. In fact, the words “oscillation” and “rhythm” are generally used to describe the subfield of neuroscience dedicated to study of the brain’s electrical activity (Buzsáki and Vöröslakos, 2023). Some neural patterns do indeed show strong periodicity. This includes well-characterized rhythms such as hippocampal theta (Vanderwolf, 1969; Winson, 1974; O’Keefe and Recce, 1993; Skaggs et al., 1996), thalamic spindles (Steriade, 2006; Niethard et al., 2018) and slow waves observed in the cortex during sleep (Steriade et al., 1993; Contreras et al., 1996; Sanchez-Vives and McCormick, 2000). Nonetheless, neuronal processes can also display non-periodic dynamics (Burns et al., 2011; Xing et al., 2012; Weber and Pillow, 2017; Naud and Sprekeler, 2018; van Ede et al., 2018; Donoghue et al., 2020; Steinmetz et al., 2021; Williams et al., 2021; Spyropoulos et al., 2022). In addition, most signals, periodic or not, have spectra where some frequencies are enhanced. Gaussian functions are non-periodic, yet exhibit a spectrum with a strong representation of low frequencies (Starosielec and Hägele, 2014). Thus, the notion that oscillations underlie changes in the spectrum of neural activity appears generally restrictive.

Here, we provide a didactic discussion for the often-intimidating interpretation of the frequency spectrum of neural recordings. Using a well-developed and broadly applicable theoretical framework (Cox and Lewis, 1966; Verveen and DeFelice, 1974; Rice, 1977; Bédard et al., 2006; Díaz et al., 2023), we show how neural patterns are usefully conceptualized as processes where discrete events can occur with varying degrees of periodicity. Using simulations, we illustrate how multiple processes can sum up in the local field potential (LFP) and how the basic properties of the Fourier transform shape the frequency spectrum of neural recordings. We show that the spectral signature of neural patterns depends essentially on 2 aspects: (1) the distribution of events in time and (2) the waveform of the voltage induced by individual events. Finally, we show examples of how this conceptual framework can be applied to decipher processes inducing changes in the spectral profile of real-world data acquired in the primary visual cortex of behaving mice.

## Materials and methods

All simulations and analysis were performed in Matlab 2018b (Mathworks). The scripts used to generate the figures are available online.^1^ Simulated time series had a sampling rate of 1 kHz and a duration of 1000 s. Event timing pulse trains had a value of 1 at the time of events and zero everywhere else. Except in the case of perfectly periodic processes, event intervals were drawn from a gamma distribution:


P(t)=1/(b*aΓ(a))*t*a-1exp(-t/b)

where P(t) is the probability density at time t, Γ (x) is the gamma function of x (i.e., the gamma function is a generalization of the factorial to real numbers), and a and b are parameters determining the shape of the distribution. The process is sub-Poissonian if *a* < 1, Poisson if *a* = 1 and super-Poissionian if *a* > 1. Parameter b was always set to 1/(a * f) where f is the overall event frequency of the process. Values of a and f for all simulations are detailed in Table 1.

**TABLE 1:** Parameters used for simulation.

Figures	Frequency	Timing	Waveform
Figure 1A	40 Hz	Super-poissonian (*a* = 0.1)	–
Figure 1B	40 Hz	Poisson (a = 1)	–
Figure 1C	40 Hz	Sub-poissonian (a = 10^1.5^)	–
Figure 1D	40 Hz	Regular	–
Figure 2A	40 Hz	Poisson (a = 1)	–
Figure 2B	40 Hz	Poisson (a = 1)	Alpha function (τ = 5.6 ms)
Figure 2C	40 Hz	Poisson (a = 1)	Morlet (σ = 1.7 ms; t0 = 3.7 ms; p = 6.7 ms)
Figure 2D	40 Hz	Poisson (a = 1)	Morlet (σ = 66.7 ms; t0 = 0 ms; p = 66.7 ms)
Figure 3A	–	White Noise	RC filter (τ = 35 ms)
Figure 3B	75 Hz	Poisson (a = 1)	Morlet (σ = 3.3 ms; t0 = 7.3 ms; p = 13.3 ms)
Supplementary Figure 1A	40 Hz	Poisson (a = 1)	–
Supplementary Figure 1B	40 Hz	Sub-poissonian (a = 10)	–
Supplementary Figure 1C	40 Hz	Sub-poissonian (a = 100)	–
Supplementary Figure 1D	40 Hz	Sub-poissonian (a = 1000)	–
Supplementary Figure 1E	40 Hz	Regular	–
Supplementary Figure 2A	50 Hz	Poisson (a = 1)	–
Supplementary Figure 2B	80 Hz	Sub-poissonian (a = 20)	–
Supplementary Figure 2C	20 Hz	Sub-poissonian (a = 40)	–
Supplementary Figure 3A	40 Hz	Regular	–
Supplementary Figure 3B	40 Hz	Regular	Alpha function (τ = 5.6 ms)
Supplementary Figure 3C	40 Hz	Regular	Morlet (σ = 1.7 ms; t0 = 3.7 ms; p = 6.7 ms)
Supplementary Figure 3D	40 Hz	Regular	Morlet (σ = 66.7 ms; t0 = 0 ms; p = 66.7 ms)
Supplementary Figure 4A	40 Hz	Sub-poissonian (a = 10^1.2^)	
Supplementary Figure 4B	40 Hz	Sub-poissonian (a = 10^1.2^)	Hann (p = 5 ms)
Supplementary Figure 4C	40 Hz	Sub-poissonian (a = 10^1.2^)	Hann (p = 25 ms)
Supplementary Figure 4D	40 Hz	Sub-poissonian (a = 10^1.2^)	Hann (p = 125 ms)
Supplementary Figure 5A	7 Hz	Sub-poissonian (a = 10^1.5^)	Hann (Inverted; p = 142.9 ms)
Supplementary Figure 5B	80 Hz (7 Hz modulated)	Sub-poissonian (a = 10^1.5^)	Hann (Inverted; p = 12.5 ms) (scaled 1/8th)

Recurring neural events were simulated by convolving event pulse trains with waveform traces of 500 ms. Various waveforms (i.e., impulse response functions) were considered. Synaptic events were modeled with the alpha function (Markram et al., 1997):


A⁢(t)=H⁢(t)*t*exp⁡(-t/tau)

Where A(t) is the alpha function at time t, H(t) is Heaviside step function, and tau is a parameter determining the time course of the synaptic response. Parameter tau was always set to 5.6 ms.

Spikes and spindles were modeled with real valued Morlet wavelets (i.e., the multiplication of a sinusoid with a gaussian window):


W(t)=exp(-(t-μ)/2(2*σ))*sin((t-t)0*(2*π)/p)

Where W(t) is the Morlet wavelet at time t, μ and σ are the mean and standard deviation of the gaussian component, t_0_ is the offset of the sine function and p is its period. Parameter μ was always set to zero (i.e., centered). Values of σ, t_0_, and p for all simulations are detailed in Table 1.

To study the effect of waveform width, waveforms were modeled as Hann window function (i.e., one cycle of a sinusoid) with varying periods p (Table 1).

Background synaptic noise was modeled by convolving white noise with a 500 ms waveform corresponding to the impulse response function of a passive resistive capacitive (RC):


F(t)RC=(1/tau)*exp(-t/tau)

Where F_RC_(t) is the value of the impulse response function of the RC filter at time t, and tau is a parameter determining the time course of the response (note the similarity to the alpha synaptic response). Parameter tau was set to 35 ms based on values inferred from recording of the membrane resistance and capacitance of excitatory neurons in the rat barrel cortex (Karagiannis et al., 2009).

The spectrum of waveforms was computed by tapering with a Hann window and taking the squared amplitude of the Fourier transform. The spectrum of recurring event time series was estimated with Welch’s method, that is by averaging the squared amplitude of the Fourier transform within non-overlapping 500 ms segments tapered with a Hann window (Percival and Walden, 1993).

The experiment described in Figure 4 is part of a data set used in Perrenoud et al. (2022). Briefly, a mouse was chronically implanted in the primary visual cortex (∼2.5 mm left and ∼4mm posterior from Bregma) with A16 probes (Neuronexus) having 16, 50 μm spaced, recording sites. The mouse was habituated to run head-fixed on a wheel and recorded in this configuration using a Digital Lynx SX system (Neuralynx). The method used for the extraction of gamma events is called CBASS and is described and freely available at (https://github.com/cardin-higley-lab/CBASS). CBASS ties increased power within a frequency band of the spectrum [here: gamma (30–80 Hz)] during a particular state (here: running) to discrete events in time. The LFP is band-pass filtered at the frequency band of interest. Candidate events are then selected at the trough of the filtered LFP in an arbitrary channel. The dynamics of candidate events across channels are parameterized and a variant of the K-means algorithm is used to find a cluster of events whose dynamics are more prevalent during the state of interest.

## Results

To understand the frequency spectrum of neural recordings, it is useful to first consider the origin of the brain’s electric field. Neurons encode signals through variations of their membrane potential which are induced by the diffusion of Na+, K+, Cl− and Ca2+ ions through tightly controlled ion channels (Buzsáki et al., 2012). This movement causes variations in the electric field whose behavior is described by Maxwell’s equations. With a perfect knowledge of the brain’s electric (and magnetic) field, Maxwell’s equations might in principle, permit to infer movements of charges at the neuronal level. However, the sensitivity of our recording methods and the complexity of ion movement through the several billions of neurons that constitute the brain make this intractable in practice.

While the brain’s electric activity can give the impression of a continuously evolving chaos, it is made out of many repeating elements. These elements are the activation of synapses, the firing of neurons or, at a larger scale, coordinated firing and synaptic barrages within one brain region, or from one area to another (Womelsdorf et al., 2014). Each such event has a defined impact on the brain’s electric field and may thus induce a voltage deflection in a recording electrode. A recurring event, such as the firing of one neuron, can have of a strong influence on a signal depending on its magnitude and its proximity (Schmitzer-Torbert et al., 2005). We will thus start by considering how one recurring event affects the spectrum.

### Impact of a recurring event’s temporal distribution on the Fourier spectrum

**FIGURE 1:**
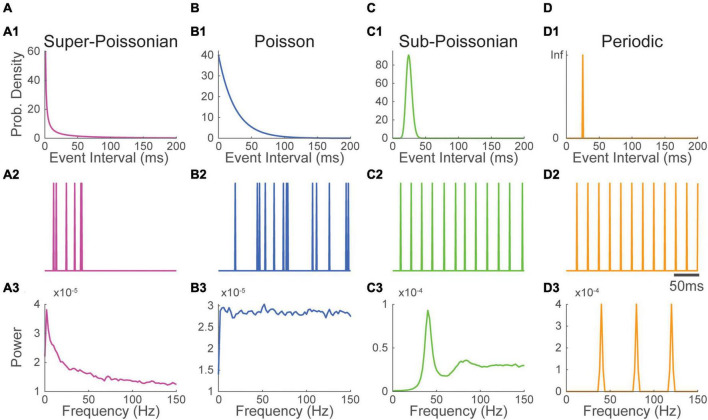
Super-Poissonian, Poisson, sub-Poissonian and periodic point-processes have recognizable Fourier spectra. (1) Event interval distribution, (2) excerpt of an event train and (3) Fourier spectrum of panel **(A)** super-Poissonian, **(B)** Poisson **(C)** sub-Poissonian and **(D)** perfectly periodic point processes. Distinct inter-event interval distributions translate into specific energy distribution in the Fourier spectrum. All processes have a mean frequency of 40 Hz.

Let us first examine the influence on the spectrum of how often and regularly an event occurs. To assess this, we will idealize recurring events and treat them as point processes (Cox and Lewis, 1966; Perkel et al., 1967). Point processes are completely defined by their inter-event interval distribution. The simplest and most commonly occurring type of point-process is the Poisson process. Poisson processes are characterized by a decaying exponential inter-event interval distribution (Figure 1B) and have 2 interesting properties: (1) the probability of an event at any given time is invariant and entirely unaffected by the process’s history; (2) the variance of the number of events per unit of time is equal to the event rate.

Point processes can be generally categorized by how regularly events occur compared to a Poisson process. Processes happening more regularly have a less variable inter-event interval. The variance of the number of events per unit of time in thus lower than the event rate. These processes are called sub-Poissonian (Figure 1C). As the variance of the event interval drops, the process begins to resemble a periodic process (Supplementary Figure 1). In the most extreme case, the variance of the inter-event interval is null, and the process is perfectly periodic (Figure 1D). Conversely, the variance of the number of events per unit of time can be superior to the event rate. In this case, the process is called super-Poissonian (Figure 1A). The firing of bursting neurons is an example of super-Poissonian process (Williams et al., 2021).

The gamma distribution (among others) can be parameterized to generate point processes (called gamma processes) having Poisson, super-Poissonian or sub-Poissonian inter-event intervals. To illustrate how the variance of point-processes affect their frequency spectrum, we parameterized gamma processes to generate super-Poissonian, Poisson and sub-Poissionian event time series. A perfectly periodic event times series was also constructed. All four time-series had a simulated sampling rate of 1000 Hz and a matched event rate of 40 Hz (Figure 1). The Fourier spectrum of each time series was then estimated using Welch’s methods with a 500 ms Hann window (Materials and Methods), a method classically used to estimate Fourier spectra in real data.

As can be seen in Figure 1B3, the spectrum of a Poisson train of event is perfectly flat. While the rate of a Poisson process affects overall power (energy per unit of time), energy remains evenly distributed across frequencies. The spectrum of a super-Poissonian gamma process is a decaying exponential and has thus more energy at low frequencies (Figure 1A3). Conversely, the spectrum of sub-poissonian gamma process shows reduced power at low frequency, a pronounced peak around the rate of the process and a flat energy distribution for higher frequencies (Figure 1C3). As the process becomes more regular secondary peaks start appearing at the harmonics of the processes rate (i.e., integer multiples of the core frequency, in our case 80 Hz, 120 Hz and so on; Supplementary Figure 1). For a perfectly periodic point process, the power is entirely concentrated and evenly distributed across the process’s rate and its harmonic (Figure 1D3). It thus appears that the event distribution of a process has a substantial and recognizable influence on the shape of its spectrum.

Periodic, Poisson and sub or super-Poissionian gamma processes are idealized processes that might not be representative of what happens in the brain. To illustrate how the timing of events affect the Fourier spectrum in more arbitrary cases, we built a compound inter-event interval distribution by adding the distributions a Poisson process and 2 sub-Poissonian gamma processes having distinct rate and variance (Supplementary Figure 2). Accordingly, the inter-event interval distribution showed two prominent modes on top of an exponential decay. We generated an event times-series following this distribution and for each summed process for comparison. The Fourier spectrum of each time-series was computed as described above.

The Fourier spectrum of the compound process (Supplementary Figure 2D3) closely resembled the sum of the Fourier spectra of the summed process (Supplementary Figures 2A3, B3, C3). Two main peaks were observed respectively at the frequencies of each sub-Poissonian process. These results illustrate that, for a point process, peaks in the spectrum are broadly indicative of modes (i.e., preferred intervals) in the inter-event interval distribution.

### Impact of a recurring event’s waveform on the Fourier spectrum

So far, we have only considered idealized time series where the impulse response function (i.e., the waveform of the voltage induced by one event) of single events is a perfect pulse (i.e., having a value of 1 at the time of the event and zero everywhere else). In actual recordings of the brain’s field activity, repeating events will have more complex waveforms. We will now consider how different waveforms affects the Fourier spectrum in conjunction with the event interval distribution. For the sake of simplicity, we will only consider that the waveform of all events in a process does not vary in shape or amplitude.

A train of recurring events can be seen as the convolution of its waveform with a signal representing the events timing such as the point-processes considered in the previous section. Such processes have a well-developed theory and are often referred to as *generalized shot noise* (Schottky, 1918; Rice, 1977; Díaz et al., 2023). The Fourier transform has the remarkable property that the spectrum of the convolution of two time series is the product of their spectrum. In other words, convolution in the time domain translate into a simple multiplication in the frequency domain (Percival and Walden, 1993). Within the accuracy of estimation methods, the Fourier spectrum of a time series of recurring events is thus equivalent to the product of the spectra of the event’s waveform and that of its event timing pulse train.

**FIGURE 2:**
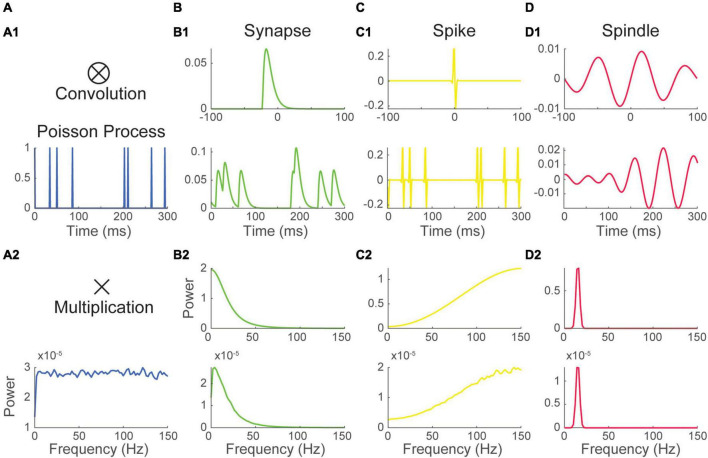
The Fourier spectrum of recurring event trains depends on the inter-event interval distribution and the waveform of single events. (1) **(A)** a Poisson process was convolved with 3 waveforms (top) mimicking the shape of panel **(B)** a synaptic event, **(C)** a spike and **(D)** a spindle, resulting in three distinct recurring event time series having the same event timing (bottom). (2) Convolution in the time domain translates into a simple multiplication into the frequency domain. Thus, the Fourier spectrum of the recurring event time series in B1, C1, and D1 (bottom) is simply the product of the spectrum of their waveform (top) and the spectrum of the Poisson pulse train (A2). Here, as Poisson processes have a flat spectrum, the spectrum of event times series is essentially determined by that of their waveform. The timing train **(A)** is identical for all processes and is not meant to be realistic.

To illustrate how this interaction shapes the Fourier spectrum, we considered three basic waveforms: 1) the alpha function, a popular choice to model synaptic responses (Figure 2B1); and 2 real-valued Gabor wavelet (the product of a sinusoidal and a gaussian window) shaped respectively to resemble 2) a spike (Figure 2C1) and 3) a spindle (Figure 2D1). All impulse response functions have specific Fourier spectra (Figures 2B2, C2, D2). The alpha function has a spectrum where energy is concentrated in lower frequencies. The spectrum of Gabor wavelets is centered on the frequency of their sinusoid component and has a bandwidth inversely proportional to the width of their gaussian window.

Each impulse response function was then convolved with a Poissonian (Figure 2) or a periodic timing pulse train (Supplementary Figure 3). The Fourier spectrum of resulting time-series was estimated as before. As predicted by theory, all constructed event time-series had spectra nearly equal to the product of that of their timing pulse train and that of their waveform. Due to the flat shape of their timing pulse train’s spectrum, Poisson distributed event time-series have a Fourier spectrum that is essentially that of their waveform (Figures 2B2, C2, D2). Thus, as exemplified in the Poissonian spikes train constructed in Figure 2C, a pronounced peak in the spectrum can be inherited solely from the shape of a recurring event’s voltage response without periodicity in its timing.

Contrasting with Poisson trains, the energy of periodic timing pulse trains is concentrated at the process’s core frequency and its harmonics. Thus, in this case, the spectrum is more generally determined by the spectrum of the timing pulse train than by that of the waveform. However, as exemplified in Supplementary Figure 3B2, some waveforms will greatly reduce the power of a periodic timing pulse train when the energy distribution of their spectrum does not overlap.

The examples above illustrate that the spectrum of a train of recurring events is shaped both by the event timing (i.e., its timing pulse train) and the impact of single events on the signal (i.e., its impulse response function or waveform). How much each shapes the spectrum varies depending on the case. One general parameter will however influence the relative contribution of one over the other, namely the duration of the waveform relative to an events overall rate of occurrence.

To illustrate this, we considered a moderately periodic sub-Poissonian pulse train (Supplementary Figure 4). The train was then convolved with Hann functions (i.e., one cycle of a sinusoid and a popular window function for spectral estimation) of increasing width relative to the processes core frequency (40 Hz). For short Hann pulses (5 times shorter the average inter-event interval, Supplementary Figure 4B), the spectrum of the resulting event times series closely resembles that of the timing pulse train. However, for longer waveforms (5 times longer the average inter-event interval, Supplementary Figure 4D), the shape of the spectrum was mostly determined by that of the waveform.

These results indicate that neural processes with short waveforms, such as action potentials, have a spectrum that tends to be influenced by their timing (i.e., timing pulse train). Conversely, neural process with a longer waveform relative to their rate of occurrence, such as synaptic events, will have a spectral imprint that tends to be determined by the waveform of single events (i.e., impulse response function).

### How do multiple neural processes sum up in the Fourier spectrum?

We have examined the Fourier spectrum of single recurring events occurring in isolation. Neural activity is composed of many such events. We will thus now consider how multiple recurring events contribute to the voltage recorded by an electrode and how they collectively shape the Fourier spectrum of neural recordings. Let us illustrate how this may happen in a simple recording configuration.

As can be derived from Gauss’s law (the first of Maxwell’s equation), the voltage deflection caused by multiple sources at an electrode is the sum of the voltage induced by each source. Thus, the voltage induced by neural events simply sums up in the local field potential. The Fourier transform of the sum of two signals is quite simply the sum of their Fourier transforms (Percival and Walden, 1993). As this summation occurs in the complex plane, signals may at times cancel each other when locked in opposite phases. However, when signals are independent, the power of their sum will approach the sum of their power (the power is the squared norm of the Fourier transform over time). Most often, multiple neural signals thus tend to add their energy distributions to the spectrum of neural recordings. The voltage deflection induced by a single source is inversely proportional to its distance to the recording electrode. Sources that are small and relatively far away from the recording site, such as synaptic events, tend to be indistinguishable and sum into a background signal. We will thus first simulate this background signal.

**FIGURE 3:**
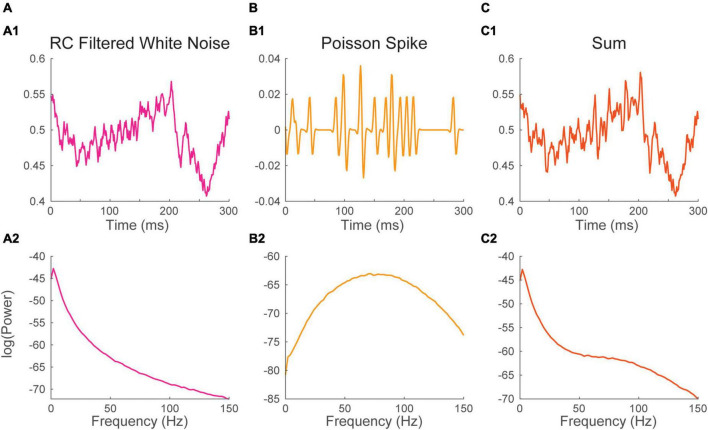
Neural processes sum their energy in the Fourier spectrum. (1) Excerpt, and (2) Fourier spectrum of panel **(A)** white noise filtered with a passive Resistive-Capacitive (RC) filter mimicking desynchronized background synaptic activity, **(B)** a Poisson spike train and **(C)** the sum of the time series in panels **(A,B)**. The spectrum of background synaptic activity in A has a characteristic 1/f^2^ shape which is inherited from the RC filter. The spectrum of the Poisson spike train in B has a bell shape peaking at 75 Hz which is inherited from the spike waveform. The spectrum of the sum of two signals approaches the sum of their spectra.

In awake cortical recordings, background synaptic events often occur at a sustained high rate, a regime that is called desynchronized (Steriade and Deschenes, 1984; Destexhe and Paré, 1999; Chance et al., 2002; Destexhe and Contreras, 2006; Haider and McCormick, 2009; Petersen and Crochet, 2013). As synaptic responses tend to be slower, the spectrum has properties that are mostly shaped by the waveform of synaptic events (i.e., impulse response function, previous section). Synaptic events display a characteristic 1/f^2^ power law energy distribution (Figure 2B2). This energy distribution is likely inherited in part from the biophysical properties of neuronal membranes which tend to behave as passive resistor-capacitor (RC) filters (Koch, 1984; Bédard et al., 2006; Freeman and Zhai, 2009), though it has received numerous interpretations (Bédard et al., 2006; Miller et al., 2009; Touboul and Destexhe, 2010; Gao et al., 2017; Donoghue et al., 2020; Schaworonkow and Voytek, 2021; O’Byrne and Jerbi, 2022). Here, we simply simulated the background LFP signal as white noise passing through an RC filter (Figure 3A1, Materials and methods). The spectrum of that times series was computed as before (Figure 3A2).

**FIGURE 4:**
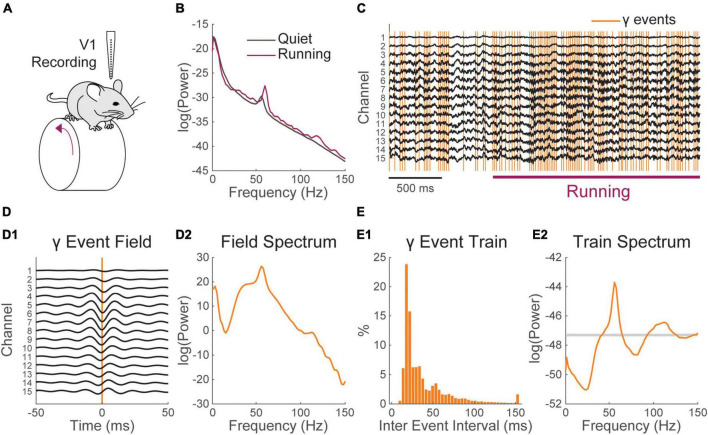
The spectrum of gamma activity in the mouse visual cortex is shaped by the timing of gamma events. **(A)** Schematic of the recording configuration. A linear electrode array is used to record the local field potential (LFP) across the layers of the primary visual cortex (V1) of a mouse freely running on a wheel. **(B)** Locomotion (purple) induces an increase in LFP power in the gamma range (30–80 Hz) having a narrow band peak around 55–60 Hz and broad band component. **(C)** Excerpt of the recording around locomotion onset. Locomotion modulated gamma activity can be tied to discrete network event [orange; further detail on the method can be found in Perrenoud et al. (2022)]. **(D)** (1) Average LFP around gamma events and (2) its Fourier spectrum. The average LFP shows a broad band and a narrow band peak in its energy distribution. **(E)** (1) Inter-event interval distribution of gamma events and (2) Fourier spectrum of the event timing train of gamma events. The gray bar in E2 represents the 95% confidence interval of the spectra of matched Poisson trains (1,000 randomizations). The broad-band and a narrow-band components of locomotion modulated gamma activity are visible in the Spectrum of the event’s timing.

Recordings of the local field potential will also often be impacted by action potentials originating from one or several neurons (i.e., units) located in the vicinity (within ∼30–50 μm) of the recording electrode (Schmitzer-Torbert et al., 2005). The voltage deflection induced by these action potentials can sometimes have a significant impact on the spectrum. To illustrate this, we simulated neuronal firing by convolving a Poisson timing pulse train with a spike shaped Morlet wavelet (Figure 3B1). As the timing of the train is Poissonian, the distribution of energy of the spectrum is purely inherited from the waveform of the Morlet spike (i.e., the impulse response function). The spectrum of the process showed a bell-shaped energy distribution with a peak at 75 Hz (Figure 3B2).

The two time-series were then summed to simulate a common recording scenario where neuronal firing at the vicinity of the electrode adds to a background synaptic signal (Figure 3C1). The spectrum of the resulting times series was estimated as before (Figure 3C2). The spectrum of the sum of the two signal is nearly equivalent to the sum of their spectra. The Poisson spike train added to the spectrum of the background LFP an induced an upward inflection of the energy distribution peaking at 75 Hz. This simulation illustrates how the contributions of multiple neuronal processes can be added up in the Fourier Spectrum. These results also show that a relatively complex spectrum can arise from the simple addition of non-periodic processes.

### Quasi-periodic processes can be modeled as recurring events

As illustrated in the example above, background synaptic activity has considerable influence on the shape of the LFP spectrum, especially at frequencies under ∼50 Hz (Buzsáki et al., 2012). We have also seen that under a desynchronized regime, the spectral energy of this background activity tends toward a 1/f^2^ distribution (Donoghue et al., 2020; Schaworonkow and Voytek, 2021). However, background synaptic activity will at times include patterns of synchronized synaptic barrages (Buzsáki et al., 2012; Buzsáki and Vöröslakos, 2023). These barrages tend to target specific subregions of the somato-dendritic compartment and thus, to induce return currents as neurons equilibrate their membrane potential. In oriented structures, such as the cortex and hippocampus, return currents add up and greatly amplify the voltage induce by barrages in the LFP (Buzsáki et al., 2012). Thus, localized synaptic barrage often have a strong influence on the spectrum.

Some well-characterized patterns of synaptic barrages show strong periodicity. These include thalamocortical spindles (Steriade and Deschenes, 1984; Timofeev et al., 1996; Steriade, 2006), the theta rhythm of the hippocampus (Vanderwolf, 1969; O’Keefe and Recce, 1993; Skaggs et al., 1996) or cortical activity during slow wave sleep (Steriade et al., 1993; Contreras et al., 1996). At times neural patterns having distinct frequencies may also interact (Canolty and Knight, 2010; Cohen, 2017). It is often natural to treat quasi-periodic or transiently periodic neural pattern as oscillations. However, we will illustrate how it is also useful and generally applicable to conceptualize these patterns as a train of recurring events such as those described in the previous sections.

The theta rhythm of the hippocampus (∼7 Hz) is remarkably regular and is generally observed during locomotion (Vanderwolf, 1969; O’Keefe and Recce, 1993; Skaggs et al., 1996). Here we modeled hippocampal theta by convolving a quasi-periodic sub-poissonian timing train having a mean frequency of 7 Hz with an inverted Hann function having a matching period (Table 1 and Supplementary Figure 5A1). The spectrum of this process shows a peak at 7 Hz and closely resemble a sine wave (Supplementary Figure 5A). Hippocampal theta activity is often accompanied by high frequency activity in the gamma range (30–80 Hz) (Bragin et al., 1995; Csicsvari et al., 2003). Gamma activity generally occurs during the downward phase of theta activity (Lisman and Jensen, 2013). This modulation of high frequencies by lower frequency activity is known as cross frequency coupling (Canolty and Knight, 2010). We model theta-nested gamma activity as a smaller inverted Hann function driven by a quasi-periodic timing train having a mean frequency of 80 Hz. Events occurring during the upward phase of theta were dropped (Supplementary Figure 5B1). The spectrum of theta-modulated gamma showed prominent peaks in the gamma range (∼80 Hz), as well as theta (7 Hz) and its first harmonic (15 Hz) (Supplementary Figure 5B2). This illustrates how high frequency processes modulated by a lower frequency display increased energy at that low frequency simply because of the longer latencies that this modulation induces in their event timing. As for the simulation displayed in Figure 3, the spectrum of the sum of theta and theta-modulated gamma closely approaches the sum of their spectra (Supplementary Figure 5C).

### Identifying recurring patterns of neural activity *in vivo*

The temporal dynamics of synaptic barrages and their influence on the spectrum tend to vary from region to region and involve specific pathways. Characterizing these patterns and their influence on neural processing is a subject of active research (Girardeau et al., 2009; Cei et al., 2014; Veit et al., 2017, 2023; Uran et al., 2022; Buzsáki and Vöröslakos, 2023). Conceptualizing neural patterns as train of recurring events can also be a powerful way to explore their properties *in vivo*. To illustrate this, we will use data that we have acquired in a recent study focusing on activity induced in the gamma range (30–80 Hz) in the visual cortex of mice (Perrenoud et al., 2022).

Gamma activity arises in many brain regions under varying behavioral contingencies (Bosman et al., 2012; Cardin, 2016; Chen et al., 2017; Uran et al., 2022; Fernandez-Ruiz et al., 2023). In the mouse visual cortex, a specific increase of gamma activity is observed during locomotion (Niell and Stryker, 2010; Vinck et al., 2015). This activity comprises a narrow band component around 60 Hz (Saleem et al., 2017; Shin et al., 2023) and a broad band component starting from ∼30 Hz and expending to high frequencies (Figure 4B). In a recent study (Perrenoud et al., 2022), we took advantage of multichannel electrode arrays to show that this spectral increase can be tied to recurring events having a specific pattern of propagation across cortical layers (Figures 4A, C, Materials and methods). Gamma events occurred more frequently during locomotion but also happened at a sustained rate during quiescence (Figure 4C). Decomposing gamma activity as an event train allows examination of how it entrains cortical neurons in relation with behavior with a high temporal resolution (Perrenoud et al., 2022) and provides some insight into how its spectral signature arises and contributes to the LFP.

Gamma events showed a complex inter-event distribution with a prominent mode around 17.8 ms, showing that gamma events tend to occur in rapid sequences or bursts (Figure 4E1). Averaging the field potential around events shows a rhythmic pattern of activity (Figure 4D1), whose spectral energy distribution resembles that observed during locomotion (taking away the 1/f^2^ RC filtering induced by neural membrane, Figure 4D2). As gamma events shift phase across recording locations (Figure 4D1), it is difficult to precisely isolate the waveform of single events. However, we can now estimate how much of the shape of cortical gamma activity is determined by event timing. To do this we computed an idealized perfect pulse times series having a value of one at the time of events and zero everywhere else. The spectrum of that time series was computed as described above. We found that the characteristic broad-band and narrow-band components of the distribution of energy of gamma activity can be derived solely by considering the events timing (Figure 4E2). To estimate the significance of this spectral distribution, we compared it to the spectrum of Poisson trains having a matching number of events (Figure 4E2, 1,000 randomizations). The broad band and narrow band gamma components of the spectrum were both well outside the 95% confidence interval of randomized trains. Gamma activity in mouse V1, is thus a real-world example of how a complex spectrum can be shaped by the temporal distribution of recurring neural events.

## Discussion

Neural activity has highly complex dynamics (Buzsaki, 2006; Buzsáki et al., 2012). The Fourier transform is a remarkably powerful tool allowing the detection of subtle changes in neural processes (Percival and Walden, 1993; Bruns, 2004). However, relating variation in the Fourier spectrum to actual neural processes is a complex problem. Thus, the interpretation of the spectrum of neural activity is rarely straightforward.

In the present study, we use a simple yet generally applicable conceptual framework to guide the interpretation of the Fourier spectrum of neural recordings (Cox and Lewis, 1966; Rice, 1977; Bédard et al., 2006; Díaz et al., 2023). We note that neural activity is made of recurring events such as synaptic currents, action potentials and, at larger scales, coordinated synaptic barrages within a region or from one region to another. This allows to model neural processes straightforwardly as the convolution of (1) a timing train and (2) a waveform modeling the voltage deflection induced by single events (Rice, 1977; Bédard et al., 2006). Using simulations, we show how the spectrum of recurring neural events depends critically on these two aspects. We then illustrate how multiple recurring events tend to sum up in the LFP and how this shapes the spectrum in a typical recording scenario. Finally, we illustrate how this framework can be used to describe the dynamics of gamma activity (30–80 Hz) in real data obtained in the mouse visual cortex (Perrenoud et al., 2022).

Increased energy in the frequency spectrum within some frequency bands is often interpreted as the reflection of oscillatory neural activity. Accordingly, neural activity is frequently studied with concepts related to oscillations such as phase, amplitude, and coherence (Vinck et al., 2023). Some neural patterns do show strong periodicity (Buzsaki, 2006; Buzsáki and Vöröslakos, 2023), including slow wave sleep cortical activity (Steriade et al., 1993; Contreras et al., 1996; Sanchez-Vives and McCormick, 2000), theta rhythm in the hippocampus (O’Keefe and Recce, 1993; Skaggs et al., 1996) or thalamic spindles (Steriade, 2006; Niethard et al., 2018). Treating these patterns as oscillatory is thus a powerful way to quantify their properties (Bruns, 2004). However, these notions will not appropriately capture the properties of non-periodic signals (Donoghue et al., 2020). When then, might it be appropriate to treat neural patterns as oscillations?

Conceptualizing neural process as recurring events can a powerful way to address this question. Recurring events can be modeled simply and straightforwardly as the convolution of a timing train and a waveform (also *called impulse response function*) (Bédard et al., 2006; Díaz et al., 2023). The theory underlying such models is well developed (Cox and Lewis, 1966; Perkel et al., 1967; Rice, 1977) and has found a wide variety of applications starting with the description of shot noise by Schottky (1918). Accordingly, such processes are often referred to as *generalized shot noise* (Rice, 1977). Here, this framework allowed us to investigate how the spectrum is shaped by processes having arbitrary degrees of periodicity. Our work indicates that a tendency toward periodicity will indeed translate into increased power within a defined frequency band. However, we also provide simple examples of how band-specific power increase can arise from process that are entirely non-periodic. This indicates that some caution is warranted when concluding that peaks in the spectrum are the signature of oscillations or rhythmicity. One must indeed make sure that there is some energy in the spectrum within a specific frequency band. However, it is important that verification be performed in the time domain. A simple check for consistency in the amplitude and phase of the filtered signal can be an appropriate way to address these concerns.

What to do then when neural signals do not show strong signs of periodicity? Here, with the example of one of our recent studies (Perrenoud et al., 2022), we illustrate how treating of neural process as recurring events can also yield key insights into their properties (Figure 4). Thinking of neural patterns as recurring events is a straightforward way to link them to tangible neural processes such as synaptic responses, action potentials, or synaptic barrages. It also permits direct quantification of how the distribution of events in time might shape the spectrum. When the waveform of the voltage induced by single events can be estimated with precision (as for action potentials) it is potentially possible to estimate the full contribution of a recurring event to the spectrum.

Identifying repeating events in the local field potential and relating them to neural processes is not a simple problem. We and others have had success in identifying recurring events by taking advantage of multichannel recordings to resolve regularity in the propagation of neural activity in space (Perrenoud et al., 2022; Sibille et al., 2022). The detection methods that we used in our case, functions on the premise that events manifest as single cycles in the filtered LFP (Perrenoud et al., 2022). Thus, it may not be broadly applicable to detect multicycle events such as spindles (Timofeev et al., 1996; Olbrich et al., 2011; Niethard et al., 2018). Further methodological development is needed in this direction. Recent progress in increasing the spatial resolution of multielectrode recordings may make this approach more feasible in the future (Jun et al., 2017; Steinmetz et al., 2021). Another important limitation of the framework introduced here is that, for the sake of clarity, we have only considered waveforms that are invariant in shape and amplitude. In a real-world scenario there might be variability in the intensity and shape of the waveform of recurring neural patterns over time. However, the simple conceptual framework introduced here may serve as a useful starting point to guide developments addressing these questions in the future.

As a final note, for the sake of brevity, we have not discussed the relationship between the spectrum of a process and its autocorrelation function (Lamarre and Raynauld, 1965; Perkel et al., 1967; Lang et al., 1999; Lévesque et al., 2020). Readers wishing to expend on the theory of spectral estimation can find extended discussion in the book ‘Spectral Analysis for Physical Applications’ (Percival and Walden, 1993).

## Data availability statement

The datasets presented in this study can be found in online repositories. The names of the repository/repositories and accession number(s) can be found below: https://doi.org/10.5061/dryad.crjdfn394.

## Ethics statement

The animal study was approved by the Institutional Animal Care and Use Committee (IACUC). The study was conducted in accordance with the local legislation and institutional requirements.

## Author contributions

QP performed the simulations and wrote the manuscript. JC acquired the funding, supervised the project, and critically reviewed the manuscript. Both authors designed the research, contributed to the article, and approved the submitted version.

## Footnotes

1
https://doi.org/10.5061/dryad.crjdfn394

## Supplementary material

Supplementary Figure 1 Transition from a Poisson to a periodic point process. (1) Event interval distribution, (2) excerpt of an event train and (3) Fourier spectrum of panel **(A)** a Poisson process, **(B–D)** sub-Poissonian processes having diminishing variance in their inter-event interval distribution and **(E)** a perfectly periodic point process. As the variance of the inter-event interval diminishes, secondary peak start to appear at the harmonics (i.e., integer multiples) of the mean frequency. All processes have a mean frequency of 40 Hz.

Supplementary Figure 2 Peaks in the spectrum of a point process reflect peaks in the event interval distribution. (1) Event interval distribution, (2) excerpt of an event train and (3) Fourier spectrum of panel **(A)** a Poisson process (mean frequency 50 Hz), **(B,C)** two sub-Poissonian processes (mean frequency, 80 and 20 Hz, respectively) and **(D)** a mixture of the processes in panels **(A–C)**. Peaks in the Fourier spectra of point processes indicate the presence of some preferred event interval.

Supplementary Figure 3 The Fourier spectrum of recurring event trains depends on the inter-event interval distribution and the waveform of single events. (1) **(A)** a perfectly periodic process was convolved with 3 waveforms (top) mimicking the shape of panel **(B)** a synaptic event, **(C)** a spike and **(D)** a spindle, resulting in three distinct recurring event time series having the same event timing (bottom). (2) Convolution in the time domain translates into a simple multiplication into the frequency domain. Thus, the Fourier spectrum of the recurring event time series in B1, C1 and D1 (bottom) is simply the product of the spectrum of their waveform (top) and the spectrum of the periodic pulse train (A2). The timing train **(A)** is identical for all processes and is not meant to be realistic. Note that the spindle waveform **(D)** and the regular time train in panel **(A)** have none overlapping spectra and cancel each other.

Supplementary Figure 4 The width of the event waveform relative to the process rate influences how much each shapes the Fourier spectrum. (1) **(A)** a quasi-periodic sub-Poissonian process was convolved with 3 Hann waveform (top) having widths **(B)** 5 time shorter, **(C)** equal, and **(D)** five time longer than the mean period of the process, resulting in three distinct recurring event time series having the same event timing (bottom). (2) Convolution in the time domain translates into a simple multiplication into the frequency domain. Thus, the Fourier spectrum of the recurring event time series in B1, C1, and D1 (bottom) is simply the product of the spectrum of their impulse response functions (top) and the spectrum of the pulse train (A2). Shorter event waveforms make the spectrum look more like that of the event timing whereas longer waveforms tend to dominate the spectrum.

Supplementary Figure 5 Complex spectra arise from cross-frequency coupling. (1) Excerpt, and (2) Fourier spectrum of panel **(A)** a 7 Hz quasi-periodic sub-poissonian event train convolved with a matched Hann window simulating hippocampal theta, **(B)** a 80 Hz train constructed similarly but where event occurrence is modulated by theta phase, and **(C)** the sum of the time series in panels **(A,B)**. Cross frequency modulation induces a clear peak in the spectrum of gamma at theta (7 Hz) and its first harmonic (15 Hz). The spectrum of the sum of two signals approaches the sum of their spectra.

## References

[B1] Bédard C. Kröger H. Destexhe A. (2006). Does the 1/f frequency scaling of brain signals reflect self-organized critical states? *Phys. Rev. Lett.* 97:118102. 10.1103/PhysRevLett.97.118102 17025932

[B2] Bosman C. A. Schoffelen J. M. Brunet N. Oostenveld R. Bastos A. M. Womelsdorf T. (2012). Attentional stimulus selection through selective synchronization between monkey visual areas. *Neuron* 75 875–888. 10.1016/j.neuron.2012.06.037 22958827 PMC3457649

[B3] Bragin A. Jandó G. Nádasdy Z. Hetke J. Wise K. Buzsáki G. (1995). Gamma (40-100 Hz) oscillation in the hippocampus of the behaving rat. *J. Neurosci.* 15(1 Pt. 1), 47–60. 10.1523/JNEUROSCI.15-01-00047.1995 7823151 PMC6578273

[B4] Bruns A. (2004). Fourier-, Hilbert- and wavelet-based signal analysis: Are they really different approaches? *J. Neurosci. Methods* 137 321–332. 10.1016/j.jneumeth.2004.03.002 15262077

[B5] Burns S. P. Xing D. Shapley R. M. (2011). Is gamma-band activity in the local field potential of V1 cortex a “clock”; or filtered noise? *J. Neurosci.* 31 9658–9664. 10.1523/JNEUROSCI.0660-11.2011 21715631 PMC3518456

[B6] Buzsaki G. (2006). *Rhythms of the brain.* Oxford: Oxford University Press.

[B7] Buzsáki G. Anastassiou C. A. Koch C. (2012). The origin of extracellular fields and currents–EEG, ECoG, LFP and spikes. *Nat. Rev. Neurosci.* 13 407–420. 10.1038/nrn3241 22595786 PMC4907333

[B8] Buzsáki G. Vöröslakos M. (2023). Brain rhythms have come of age. *Neuron* 111 922–926. 10.1016/j.neuron.2023.03.018 37023714 PMC10793242

[B9] Canolty R. T. Knight R. T. (2010). The functional role of cross-frequency coupling. *Trends Cogn. Sci.* 14 506–515. 10.1016/j.tics.2010.09.001 20932795 PMC3359652

[B10] Cardin J. A. (2016). Snapshots of the brain in action*:* Local circuit operations through the lens of γ oscillations. *J. Neurosci.* 36 10496–10504. 10.1523/JNEUROSCI.1021-16.2016 27733601 PMC5059425

[B11] Cei A. Girardeau G. Drieu C. Kanbi K. E. Zugaro M. (2014). Reversed theta sequences of hippocampal cell assemblies during backward travel. *Nat. Neurosci.* 17 719–724. 10.1038/nn.3698 24667574

[B12] Chance F. S. Abbott L. F. Reyes A. D. (2002). Gain modulation from background synaptic input. *Neuron* 35 773–782. 10.1016/s0896-6273(02)00820-6 12194875

[B13] Chen G. Zhang Y. Li X. Zhao X. Ye Q. Lin Y. (2017). Distinct inhibitory circuits orchestrate cortical beta and gamma band oscillations. *Neuron* 96 1403–1418.e6. 10.1016/j.neuron.2017.11.033 29268099 PMC5864125

[B14] Cohen M. X. (2017). Multivariate cross-frequency coupling via generalized eigendecomposition. *Elife* 6:e21792. 10.7554/eLife.21792 28117662 PMC5262375

[B15] Contreras D. Timofeev I. Steriade M. (1996). Mechanisms of long-lasting hyperpolarizations underlying slow sleep oscillations in cat corticothalamic networks. *J. Physiol.* 494(Pt. 1)(Pt. 1) 251–264. 10.1113/jphysiol.1996.sp021488 8814619 PMC1160627

[B16] Cox D. R. Lewis P. A. W. (1966). *The statistical analysis of series of events.* Netherlands: Springer.

[B17] Csicsvari J. Jamieson B. Wise K. D. Buzsáki G. (2003). Mechanisms of gamma oscillations in the hippocampus of the behaving rat. *Neuron* 37 311–322. 10.1016/s0896-6273(02)01169-8 12546825

[B18] Destexhe A. Contreras D. (2006). Neuronal computations with stochastic network states. *Science* 314 85–90. 10.1126/science.1127241 17023650

[B19] Destexhe A. Paré D. (1999). Impact of network activity on the integrative properties of neocortical pyramidal neurons in vivo. *J. Neurophysiol.* 81 1531–1547. 10.1152/jn.1999.81.4.1531 10200189

[B20] Díaz J. Ando H. Han G. Malyshevskaya O. Hayashi X. Letelier J. (2023). Recovering arrhythmic EEG transients from their stochastic interference. *arXiv* [Preprint] 10.48550/arXiv.2303.07683 42230781

[B21] Donoghue T. Haller M. Peterson E. J. Varma P. Sebastian P. Gao R. (2020). Parameterizing neural power spectra into periodic and aperiodic components. *Nat. Neurosci.* 23 1655–1665. 10.1038/s41593-020-00744-x 33230329 PMC8106550

[B22] Fernandez-Ruiz A. Sirota A. Lopes-Dos-Santos V. Dupret D. (2023). Over and above frequency: Gamma oscillations as units of neural circuit operations. *Neuron* 111 936–953. 10.1016/j.neuron.2023.02.026 37023717 PMC7614431

[B23] Freeman W. J. Zhai J. (2009). Simulated Power Spectral Density (PSD) of background electrocorticogram (ECoG). *Cogn. Neurodyn.* 3 97–103. 10.1007/s11571-008-9064-y 19003455 PMC2645494

[B24] Fries P. Reynolds J. H. Rorie A. E. Desimone R. (2001). Modulation of oscillatory neuronal synchronization by selective visual attention. *Science* 291 1560–1563. 10.1126/science.1055465 11222864

[B25] Gao R. Peterson E. J. Voytek B. (2017). Inferring synaptic excitation/inhibition balance from field potentials. *Neuroimage* 158 70–78. 10.1016/j.neuroimage.2017.06.078 28676297

[B26] Girardeau G. Benchenane K. Wiener S. I. Buzsáki G. Zugaro M. B. (2009). Selective suppression of hippocampal ripples impairs spatial memory. *Nat. Neurosci.* 12 1222–1223. 10.1038/nn.2384 19749750

[B27] Gray C. M. König P. Engel A. K. Singer W. (1989). Oscillatory responses in cat visual cortex exhibit inter-columnar synchronization which reflects global stimulus properties. *Nature* 338 334–337. 10.1038/338334a0 2922061

[B28] Haider B. McCormick D. A. (2009). Rapid neocortical dynamics: Cellular and network mechanisms. *Neuron* 62 171–189. 10.1016/j.neuron.2009.04.008 19409263 PMC3132648

[B29] Jun J. J. Steinmetz N. A. Siegle J. H. Denman D. J. Bauza M. Barbarits B. (2017). Fully integrated silicon probes for high-density recording of neural activity. *Nature* 551 232–236. 10.1038/nature24636 29120427 PMC5955206

[B30] Karagiannis A. Gallopin T. Dávid C. Battaglia D. Geoffroy H. Rossier J. (2009). Classification of NPY-expressing neocortical interneurons. *J. Neurosci.* 29 3642–3659. 10.1523/JNEUROSCI.0058-09.2009 19295167 PMC2750888

[B31] Koch C. (1984). Cable theory in neurons with active, linearized membranes. *Biol. Cybern.* 50 15–33. 10.1007/BF00317936 6324889

[B32] Lamarre Y. Raynauld J. P. (1965). Rhythmic firing in the spontaneous activity of centrally located neurons. a method of analysis. *Electroencephalogr. Clin. Neurophysiol.* 18 87–90. 10.1016/0013-4694(65)90152-5 14255033

[B33] Lang E. J. Sugihara I. Welsh J. P. Llinás R. (1999). Patterns of spontaneous purkinje cell complex spike activity in the awake rat. *J. Neurosci.* 19 2728–2739. 10.1523/JNEUROSCI.19-07-02728.1999 10087085 PMC6786059

[B34] Lévesque M. Gao H. Southward C. Langlois J. M. Léna C. Courtemanche R. (2020). Cerebellar cortex 4-12 Hz oscillations and unit phase relation in the awake rat. *Front. Syst. Neurosci.* 14:475948. 10.3389/fnsys.2020.475948 33240052 PMC7683574

[B35] Lisman J. E. Jensen O. (2013). The theta-gamma neural code. *Neuron* 77 1002–1016. 10.1016/j.neuron.2013.03.007 23522038 PMC3648857

[B36] Markram H. Lübke J. Frotscher M. Sakmann B. (1997). Regulation of synaptic efficacy by coincidence of postsynaptic APs and EPSPs. *Science* 275 213–215. 10.1126/science.275.5297.213 8985014

[B37] Miller K. J. Sorensen L. B. Ojemann J. G. den Nijs M. (2009). Power-law scaling in the brain surface electric potential. *PLoS Comput. Biol.* 5:e1000609. 10.1371/journal.pcbi.1000609 20019800 PMC2787015

[B38] Naud R. Sprekeler H. (2018). Sparse bursts optimize information transmission in a multiplexed neural code. *Proc. Natl. Acad. Sci. U.S.A.* 115 E6329–E6338. 10.1073/pnas.1720995115 29934400 PMC6142200

[B39] Niell C. M. Stryker M. P. (2010). Modulation of visual responses by behavioral state in mouse visual cortex. *Neuron* 65 472–479. 10.1016/j.neuron.2010.01.033 20188652 PMC3184003

[B40] Niethard N. Ngo H. V. Ehrlich I. Born J. (2018). Cortical circuit activity underlying sleep slow oscillations and spindles. *Proc. Natl. Acad. Sci. U.S.A.* 115 E9220–E9229. 10.1073/pnas.1805517115 30209214 PMC6166829

[B41] O’Byrne J. Jerbi K. (2022). How critical is brain criticality? *Trends Neurosci.* 45 820–837. 10.1016/j.tins.2022.08.007 36096888

[B42] O’Keefe J. Recce M. L. (1993). Phase relationship between hippocampal place units and the EEG theta rhythm. *Hippocampus* 3 317–330. 10.1002/hipo.450030307 8353611

[B43] Olbrich E. Claussen J. C. Achermann P. (2011). The multiple time scales of sleep dynamics as a challenge for modelling the sleeping brain. *Philos. Trans. A. Math. Phys. Eng. Sci.* 369 3884–3901. 10.1098/rsta.2011.0082 21893533

[B44] Penttonen M. Buzsáki G. (2003). Natural logarithmic relationship between brain oscillators. *Thalamus Relat. Syst.* 2 145–152. 10.1017/S1472928803000074

[B45] Percival D. B. Walden A. T. (1993). *Spectral analysis for physical applications.* Cambridge: Cambridge University Press. 10.1017/CBO9780511622762

[B46] Perkel D. H. Gerstein G. L. Moore G. P. (1967). Neuronal spike trains and stochastic point processes. I. The single spike train. *Biophys. J.* 7 391–418. 10.1016/S0006-3495(67)86596-2 4292791 PMC1368068

[B47] Perrenoud Q. de O. Fonseca A. H. Airhart A. Bonanno J. Mao R. Cardin J. A. (2022). Flexible perceptual encoding by discrete gamma events. *bioRxiv [Prerpint]* 10.1101/2022.05.13.491832 PMC12657229 41062693

[B48] Petersen C. C. Crochet S. (2013). Synaptic computation and sensory processing in neocortical layer 2/3. *Neuron* 78 28–48. 10.1016/j.neuron.2013.03.020 23583106

[B49] Rice J. (1977). On generalized shot noise. *Adv. Appl. Probabil.* 9 553–565. 10.2307/1426114

[B50] Saleem A. B. Lien A. D. Krumin M. Haider B. Rosón M. R. Ayaz A. (2017). Subcortical source and modulation of the narrowband gamma oscillation in mouse visual cortex. *Neuron* 93 315–322. 10.1016/j.neuron.2016.12.028 28103479 PMC5263254

[B51] Sanchez-Vives M. V. McCormick D. A. (2000). Cellular and network mechanisms of rhythmic recurrent activity in neocortex. *Nat. Neurosci.* 3 1027–1034. 10.1038/79848 11017176

[B52] Schaworonkow N. Voytek B. (2021). Longitudinal changes in aperiodic and periodic activity in electrophysiological recordings in the first seven months of life. *Dev. Cogn. Neurosci.* 47:100895. 10.1016/j.dcn.2020.100895 33316695 PMC7734223

[B53] Schmitzer-Torbert N. Jackson J. Henze D. Harris K. Redish A. D. (2005). Quantitative measures of cluster quality for use in extracellular recordings. *Neuroscience* 131 1–11. 10.1016/j.neuroscience.2004.09.066 15680687

[B54] Schottky W. (1918). Über spontane Stromschwankungen in verschiedenen Elektrizitätsleitern. *Ann. Phys.* 362:541. 10.1002/andp.19183622304

[B55] Shin D. Peelman K. Lien A. D. Del Rosario J. Haider B. (2023). Narrowband gamma oscillations propagate and synchronize throughout the mouse thalamocortical visual system. *Neuron* 111 1076–1085.e8. 10.1016/j.neuron.2023.03.006 37023711 PMC10112544

[B56] Sibille J. Gehr C. Benichov J. I. Balasubramanian H. Teh K. L. Lupashina T. (2022). High-density electrode recordings reveal strong and specific connections between retinal ganglion cells and midbrain neurons. *Nat. Commun.* 13:5218. 10.1038/s41467-022-32775-2 36064789 PMC9445019

[B57] Skaggs W. E. McNaughton B. L. Wilson M. A. Barnes C. A. (1996). Theta phase precession in hippocampal neuronal populations and the compression of temporal sequences. *Hippocampus* 6 149–172. 10.1002/(SICI)1098-106319966:2<149::AID-HIPO6>3.0.CO;2-K 8797016

[B58] Spyropoulos G. Saponati M. Dowdall J. R. Schölvinck M. L. Bosman C. A. Lima B. (2022). Spontaneous variability in gamma dynamics described by a damped harmonic oscillator driven by noise. *Nat. Commun.* 13:2019. 10.1038/s41467-022-29674-x 35440540 PMC9018758

[B59] Starosielec S. Hägele D. (2014). Discrete-time windows with minimal RMS bandwidth for given RMS temporal width. *Signal Process.* 102 240–246. 10.1016/j.sigpro.2014.03.033

[B60] Steinmetz N. A. Aydin C. Lebedeva A. Okun M. Pachitariu M. Bauza M. (2021). Neuropixels 2.0: A miniaturized high-density probe for stable, long-term brain recordings. *Science* 372:eabf4588. 10.1126/science.abf4588 33859006 PMC8244810

[B61] Steriade M. (2006). Grouping of brain rhythms in corticothalamic systems. *Neuroscience* 137 1087–1086. 10.1016/j.neuroscience.2005.10.029 16343791

[B62] Steriade M. Deschenes M. (1984). The thalamus as a neuronal oscillator. *Brain Res.* 320 1–63. 10.1016/0165-0173(84)90017-1 6440659

[B63] Steriade M. Nuñez A. Amzica F. (1993). A novel slow. *J. Neurosci.* 13 3252–3265. 10.1523/JNEUROSCI.13-08-03252.1993 8340806 PMC6576541

[B64] Timofeev I. Contreras D. Steriade M. (1996). Synaptic responsiveness of cortical and thalamic neurones during various phases of slow sleep oscillation in cat. *J. Physiol.* 494(Pt. 1)(Pt. 1) 265–278. 10.1113/jphysiol.1996.sp021489 8814620 PMC1160628

[B65] Touboul J. Destexhe A. (2010). Can power-law scaling and neuronal avalanches arise from stochastic dynamics? *PLoS One* 5:e8982. 10.1371/journal.pone.0008982 20161798 PMC2820096

[B66] Uran C. Peter A. Lazar A. Barnes W. Klon-Lipok J. Shapcott K. A. (2022). Predictive coding of natural images by V1 firing rates and rhythmic synchronization. *Neuron* 110 1240–1257.e8. 10.1016/j.neuron.2022.01.002 35120628 PMC8992798

[B67] van Ede F. Quinn A. J. Woolrich M. W. Nobre A. C. (2018). Neural oscillations: Sustained rhythms or transient burst-events? *Trends Neurosci.* 41 415–417. 10.1016/j.tins.2018.04.004 29739627 PMC6024376

[B68] Vanderwolf C. H. (1969). Hippocampal electrical activity and voluntary movement in the rat. *Electroencephalogr. Clin. Neurophysiol.* 26 407–418. 10.1016/0013-4694(69)90092-3 4183562

[B69] Veit J. Hakim R. Jadi M. P. Sejnowski T. J. Adesnik H. (2017). Cortical gamma band synchronization through somatostatin interneurons. *Nat. Neurosci.* 20 951–959. 10.1038/nn.4562 28481348 PMC5511041

[B70] Veit J. Handy G. Mossing D. P. Doiron B. Adesnik H. (2023). Cortical VIP neurons locally control the gain but globally control the coherence of gamma band rhythms. *Neuron* 111 405–417.e5. 10.1016/j.neuron.2022.10.036 36384143 PMC9898108

[B71] Verveen A. A. DeFelice L. J. (1974). Membrane noise. *Prog. Biophys. Mol. Biol.* 28 189–265. 10.1016/0079-6107(74)90019-4 4617247

[B72] Vinck M. Batista-Brito R. Knoblich U. Cardin J. A. (2015). Arousal and locomotion make distinct contributions to cortical activity patterns and visual encoding. *Neuron* 86 740–754. 10.1016/j.neuron.2015.03.028 25892300 PMC4425590

[B73] Vinck M. Uran C. Spyropoulos G. Onorato I. Broggini A. Schneider M. (2023). Principles of large-scale neural interactions. *Neuron* 111 987–1002. 10.1016/j.neuron.2023.03.015 37023720

[B74] Weber A. I. Pillow J. W. (2017). Capturing the dynamical repertoire of single neurons with generalized linear models. *arXiv* [Preprint]. 10.48550/arXiv.1602.07389 28957020

[B75] Williams E. Payeur A. Gidon A. Naud R. (2021). Neural burst codes disguised as rate codes. *Sci. Rep.* 11:15910. 10.1038/s41598-021-95037-z 34354118 PMC8342467

[B76] Winson J. (1974). Patterns of hippocampal theta rhythm in the freely moving rat. *Electroencephalogr. Clin. Neurophysiol.* 36 291–301. 10.1016/0013-4694(74)90171-0 4130608

[B77] Womelsdorf T. Valiante T. A. Sahin N. T. Miller K. J. Tiesinga P. (2014). Dynamic circuit motifs underlying rhythmic gain control, gating and integration. *Nat. Neurosci.* 17 1031–1039. 10.1038/nn.3764 25065440

[B78] Xing D. Shen Y. Burns S. Yeh C. I. Shapley R. Li W. (2012). Stochastic generation of gamma-band activity in primary visual cortex of awake and anesthetized monkeys. *J. Neurosci.* 32 13873a–13880a. 10.1523/JNEUROSCI.5644-11.2012 23035096 PMC3752128

