# Supplementary figures and images for: Beyond rhythm – a framework for understanding the frequency spectrum of neural activity
Source: Front Syst Neurosci. 2023 Aug 31;17:1217170. doi: 10.3389/fnsys.2023.1217170 (PMC10500127; doi:10.3389/fnsys.2023.1217170)

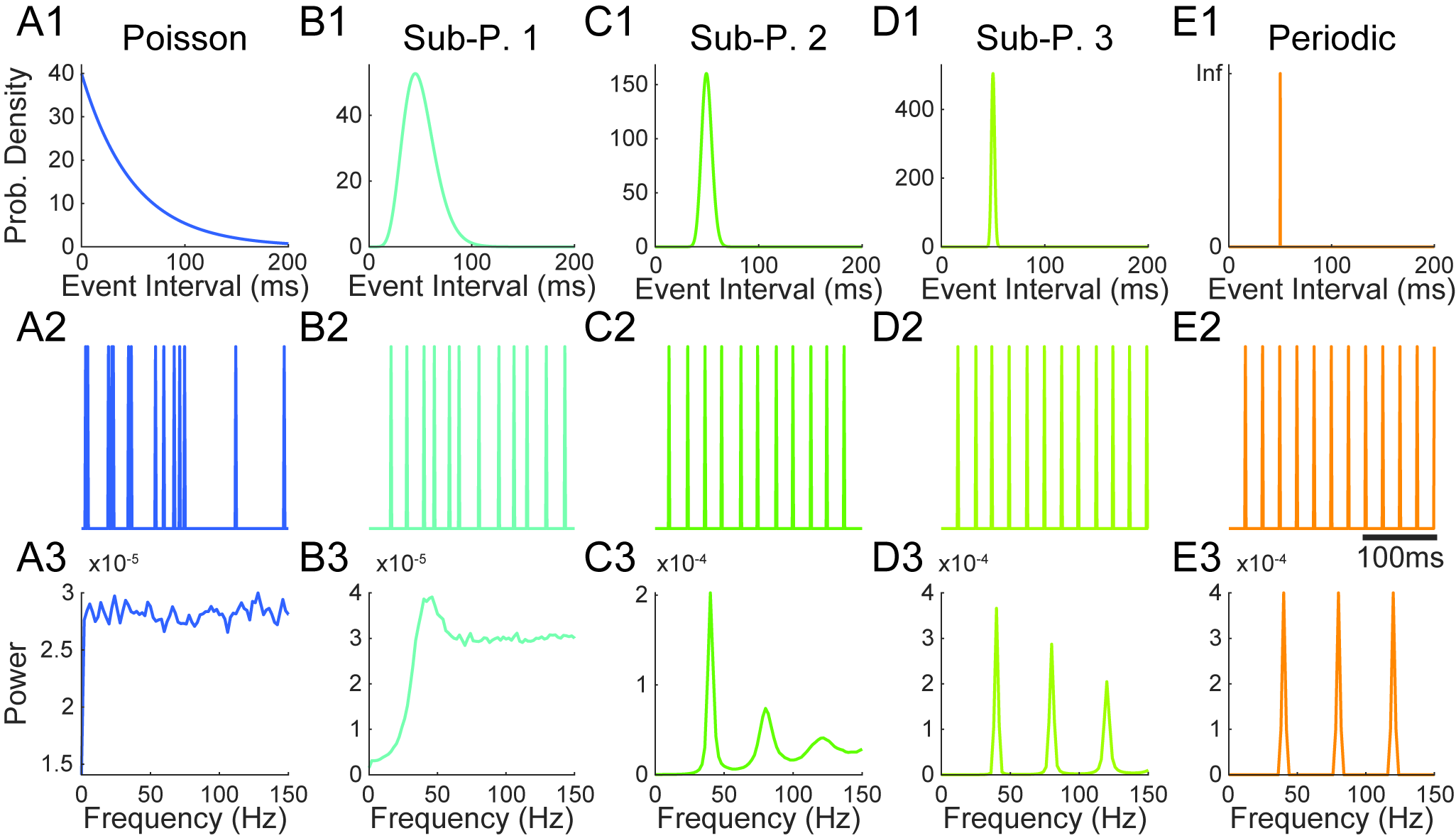

Supplement: Supplementary Figure 1 — Transition from a Poisson to a periodic point process. (1) Event interval distribution, (2) excerpt of an event train and (3) Fourier spectrum of panel (A) a Poisson process, (B–D) sub-Poissonian processes having diminishing variance in their inter-event interval distribution and (E) a perfectly periodic point process. As the variance of the inter-event interval diminishes, secondary peak start to appear at the harmonics (i.e., integer multiples) of the mean frequency. All processes have a mean frequency of 40 Hz. [file Image_1.TIF]

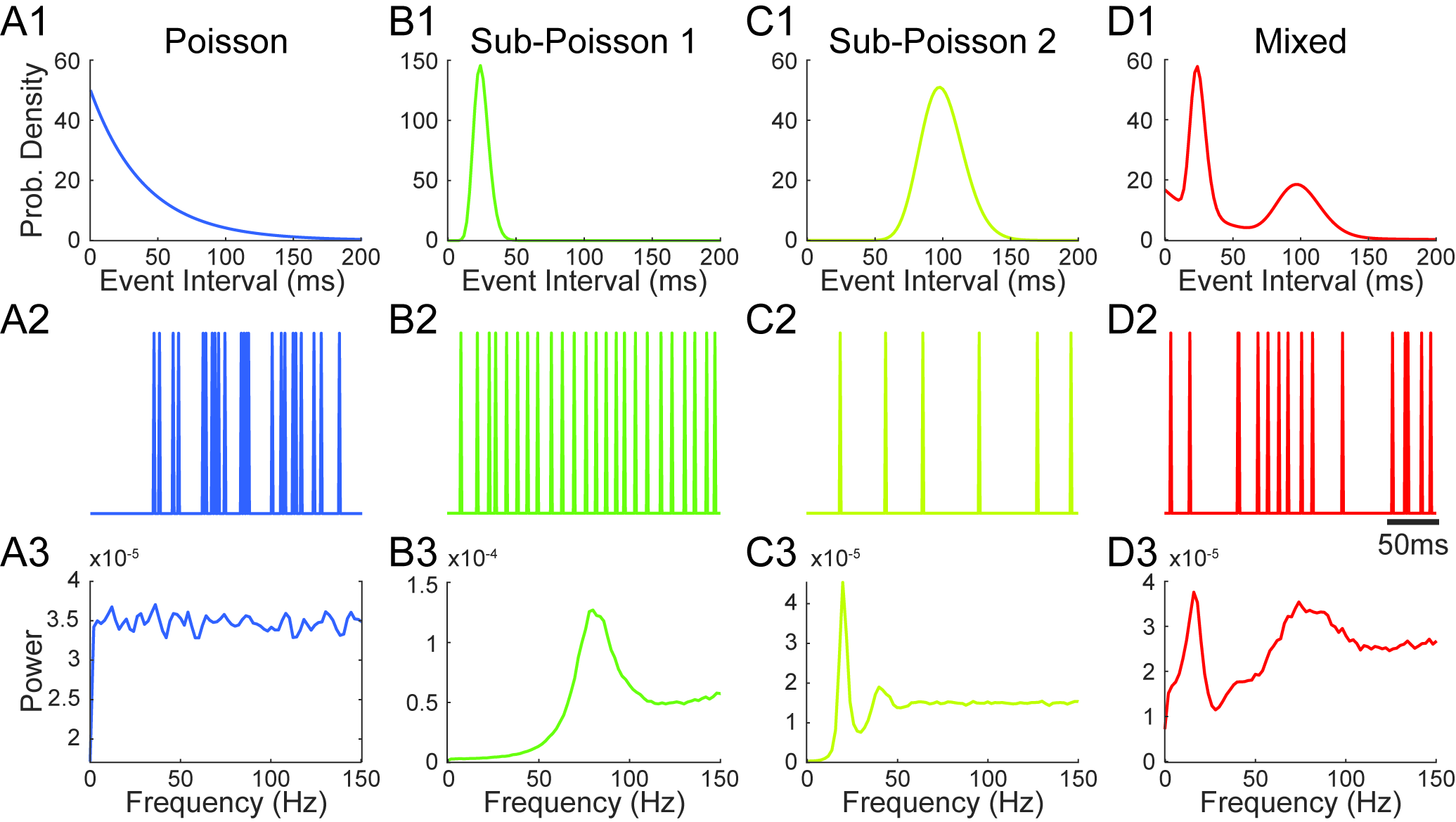

Supplement: Supplementary Figure 2 — Peaks in the spectrum of a point process reflect peaks in the event interval distribution. (1) Event interval distribution, (2) excerpt of an event train and (3) Fourier spectrum of panel (A) a Poisson process (mean frequency 50 Hz), (B,C) two sub-Poissonian processes (mean frequency, 80 and 20 Hz, respectively) and (D) a mixture of the processes in panels (A–C). Peaks in the Fourier spectra of point processes indicate the presence of some preferred event interval. [file Image_2.TIF]

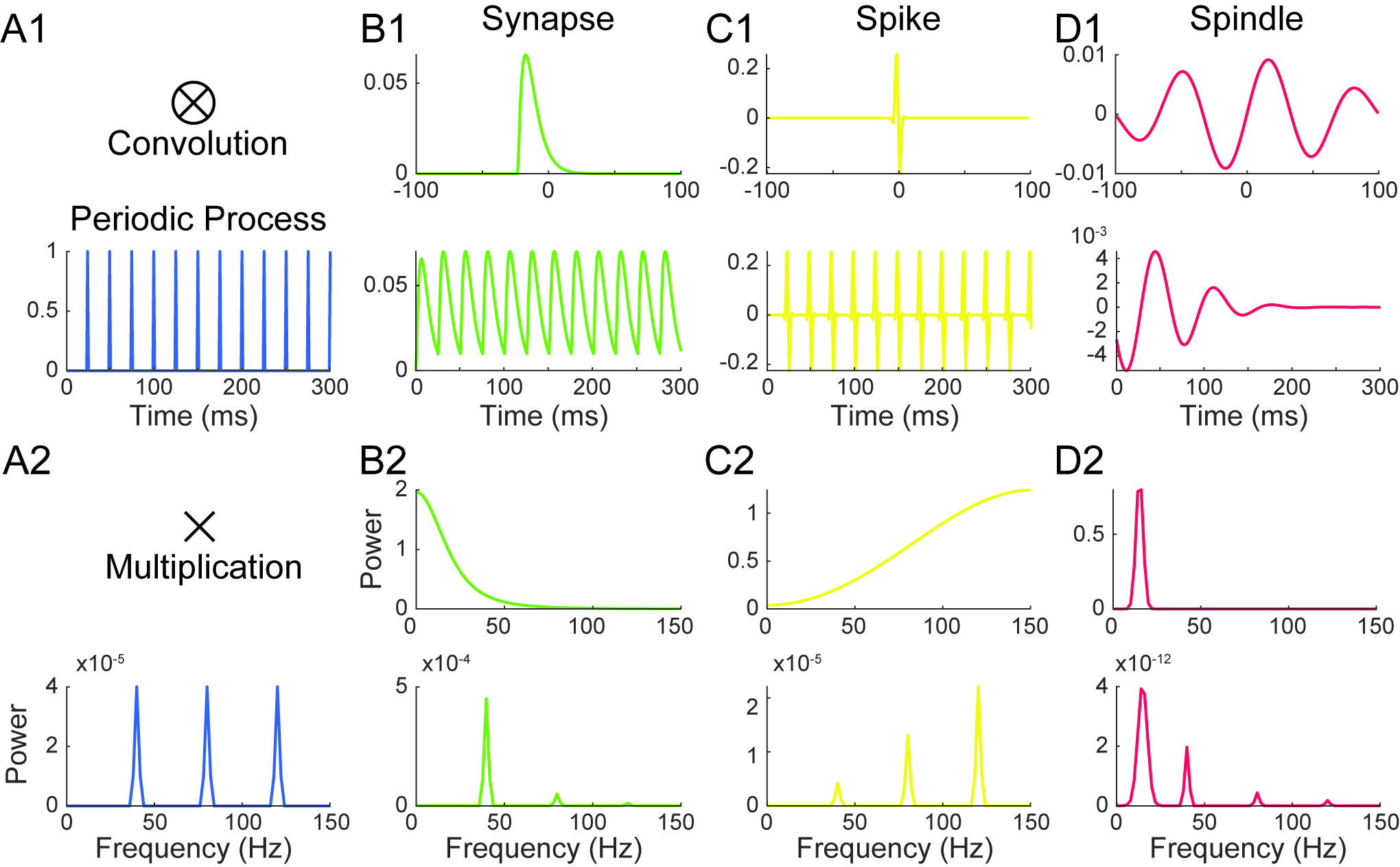

Supplement: Supplementary Figure 3 — The Fourier spectrum of recurring event trains depends on the inter-event interval distribution and the waveform of single events. (1) (A) a perfectly periodic process was convolved with 3 waveforms (top) mimicking the shape of panel (B) a synaptic event, (C) a spike and (D) a spindle, resulting in three distinct recurring event time series having the same event timing (bottom). (2) Convolution in the time domain translates into a simple multiplication into the frequency domain. Thus, the Fourier spectrum of the recurring event time series in B1, C1 and D1 (bottom) is simply the product of the spectrum of their waveform (top) and the spectrum of the periodic pulse train (A2). The timing train (A) is identical for all processes and is not meant to be realistic. Note that the spindle waveform (D) and the regular time train in panel (A) have none overlapping spectra and cancel each other. [file Image_3.TIF]

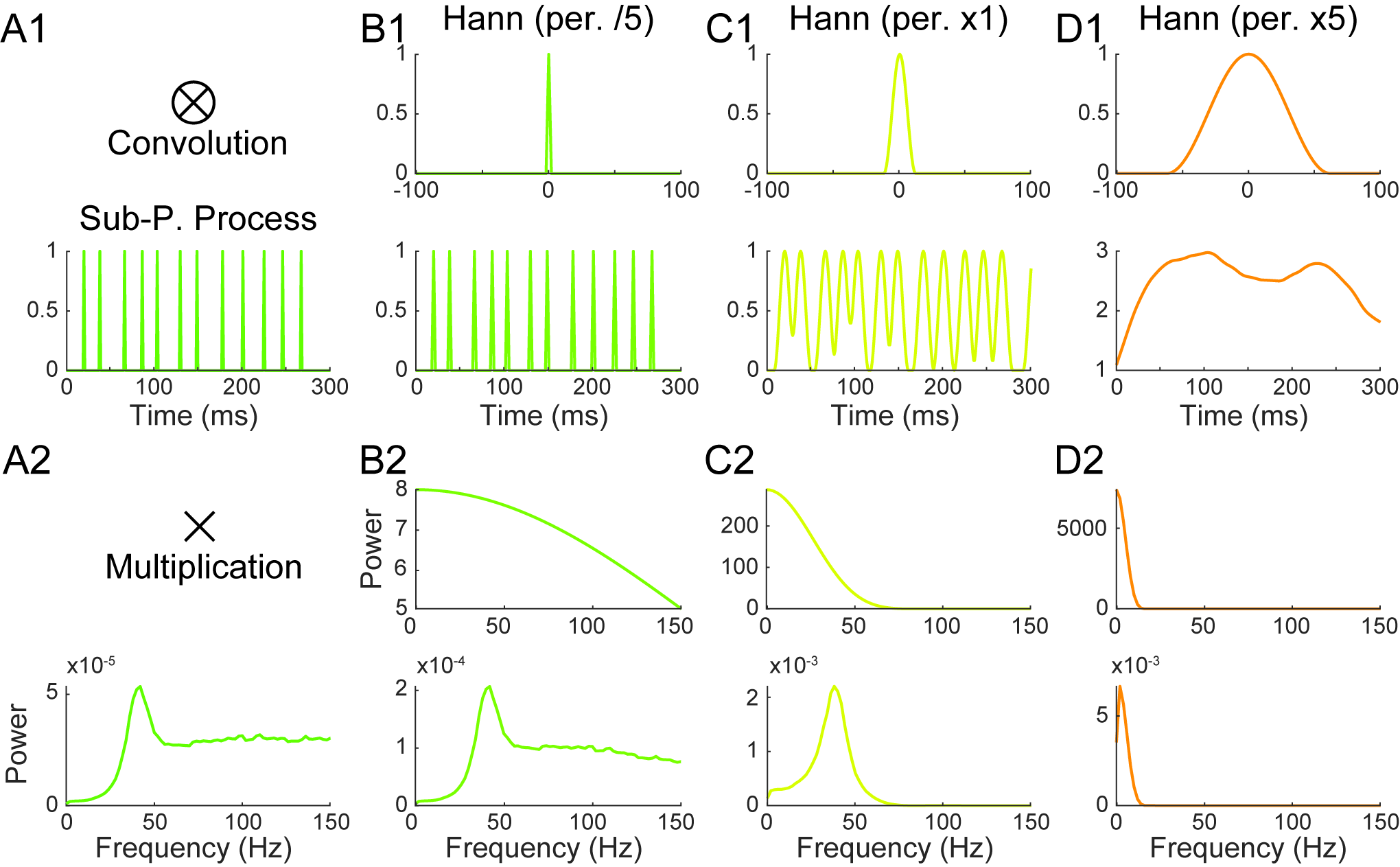

Supplement: Supplementary Figure 4 — The width of the event waveform relative to the process rate influences how much each shapes the Fourier spectrum. (1) (A) a quasi-periodic sub-Poissonian process was convolved with 3 Hann waveform (top) having widths (B) 5 time shorter, (C) equal, and (D) five time longer than the mean period of the process, resulting in three distinct recurring event time series having the same event timing (bottom). (2) Convolution in the time domain translates into a simple multiplication into the frequency domain. Thus, the Fourier spectrum of the recurring event time series in B1, C1, and D1 (bottom) is simply the product of the spectrum of their impulse response functions (top) and the spectrum of the pulse train (A2). Shorter event waveforms make the spectrum look more like that of the event timing whereas longer waveforms tend to dominate the spectrum. [file Image_4.TIF]

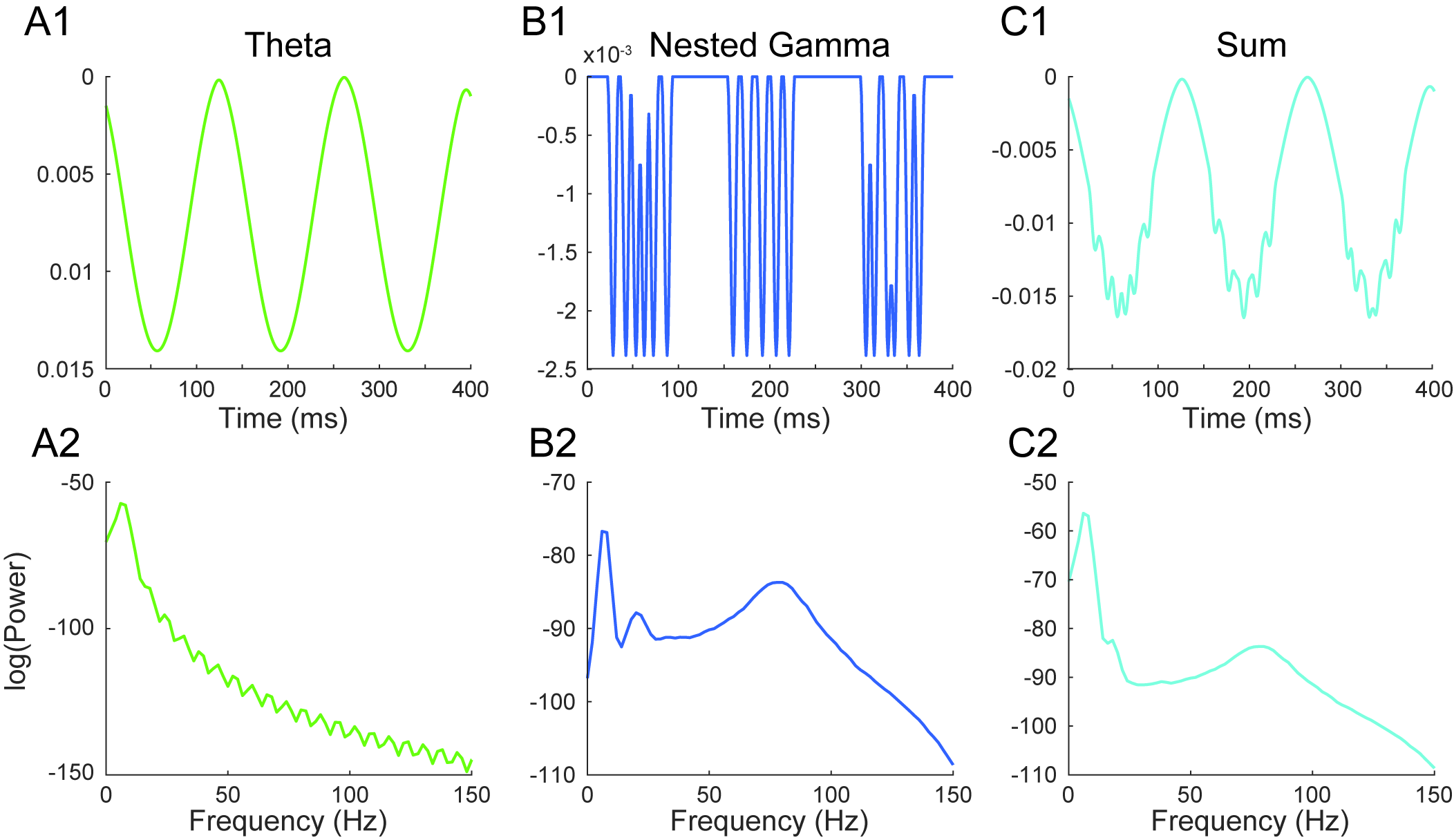

Supplement: Supplementary Figure 5 — Complex spectra arise from cross-frequency coupling. (1) Excerpt, and (2) Fourier spectrum of panel (A) a 7 Hz quasi-periodic sub-poissonian event train convolved with a matched Hann window simulating hippocampal theta, (B) a 80 Hz train constructed similarly but where event occurrence is modulated by theta phase, and (C) the sum of the time series in panels (A,B). Cross frequency modulation induces a clear peak in the spectrum of gamma at theta (7 Hz) and its first harmonic (15 Hz). The spectrum of the sum of two signals approaches the sum of their spectra. [file Image_5.TIF]
